# Targeting cancer: tumor-specific splicing events give rise to immunogenic, tumor-wide neoantigens

**DOI:** 10.1038/s41392-025-02237-4

**Published:** 2025-05-05

**Authors:** Nils Kosiol, Annkristin Heine, Peter Brossart

**Affiliations:** 1 Department of Oncology, Hematology, Rheumatology and Immune-Oncology, University Hospital Bonn, Bonn, Germany; 2 Clinical Division of Hematology, Department of Internal Medicine, Medical University of Graz, Graz, Austria

**Keywords:** Tumour biomarkers, Tumour immunology

**Fig. 1:**
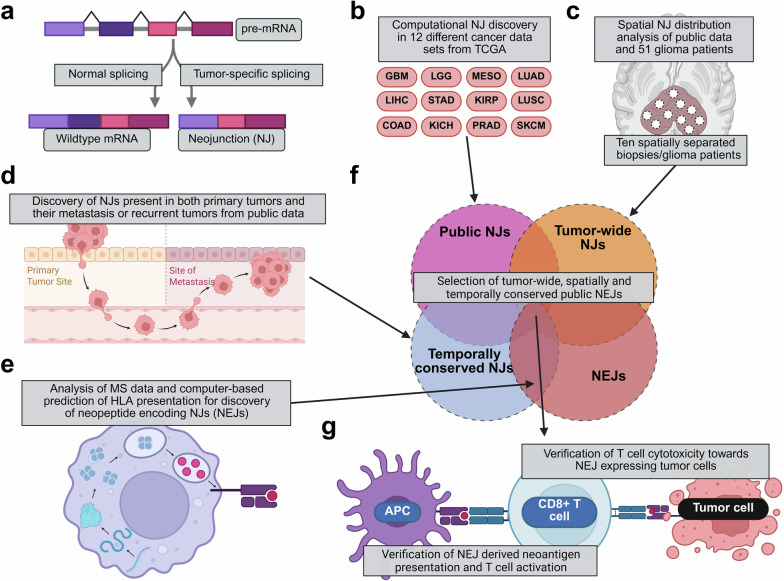
**a** Schematic overview of an example for a neojunction. **b–g** Work process that led to the discovery of public, tumor-wide, NJ-derived immunogenic neoantigens. **b** shows the 12 tumors that were investigated from the TCGA data sets. GBM glioblastoma, LGG low-grade glioma, MESO mesothelioma, LUAD lung adenocarcinoma, LIHC liver hepatocellular carcinoma, STAD stomach adenocarcinoma, KIRP kidney renal papillary cell carcinoma, LUSC lung squamous cell carcinoma, COAD colon adenocarcinoma, KICH Kidney chromophobe, PRAD Prostate adenocarcinoma, SKCM skin cutaneous melanoma. **c** Investigation of spatial distribution of public NJs was done by analyzing existing data sets and acquiring 10 spatially separated biopsies from 51 glioma patients. **d** Temporal conversation was investigated in data sets of recurring tumors and metastases. **e** NJ expression and HLA presentation were investigated using pre-existing MS data sets and bioinformatical prediction. **f** NJs that were public, tumor-wide, expressed and temporally conserved were selected for immunogenicity. **g** Induction of immunity by NEJs was confirmed with APC-based T cell activation and subsequent tumor cell killing by NEJ-targeting T cells. The figure was created with Biorender

In a recent study published in *Nature*,^1^ Kwok et al. identified tumor-wide antigens that derived from tumor-specific splicing events, known as neojunctions (NJs) (Fig. 1a). The study identified two distinct neopeptide-encoding NJs (NEJs) that were spatially and temporally conserved in glioblastoma (GBM) patients and induced an HLA-dependent T cell response. The discovery of these NEJs, as well as the pipeline used for their identification, harbors significant potential for the development of tumor vaccines and adoptive cell therapies that might be effective across various cancer entities.

Immunotherapies have transformed the landscape of cancer therapy. However, treatment efficacy is often limited by the scarcity of tumor-wide and tumor-specific antigens.^2^ While certain cancers, such as B cell lymphoma, possess well-defined, targetable antigens (e.g., CD19), many other cancers either lack tumor-specific antigens due to low mutational burden (e.g. acute myeloid leukemia)^3^ or exhibit high intratumoral heterogeneity (ITH) (e.g. GBM),^4^ meaning that neoantigens are expressed only in a subset of tumor cells. In such cases, targeted vaccines or adoptive cell therapies are often rendered ineffective.

Previous studies have successfully explored the use of NJs for the identification of tumor-specific neoantigens.^5^ However, a comprehensive analysis of the spatial distribution and temporal consistency of NJ-derived neoantigens is still lacking. Kwok et al. addressed this gap by identifying NEJs that are spatially conserved and expressed across multiple patients and tumor types. Initially, the authors analyzed publicly available datasets from The Cancer Genome Atlas (TCGA) (Fig. 1b). They demonstrated that NJs are present in 12 different tumor types and that some NJs are public, meaning that they are present across multiple tumor entities. To further investigate ITH in the context of NJs, the authors examined multi-site intratumoral sampling data from four tumor types and additionally analyzed spatial NJ distribution in 51 glioma patients by obtaining 10 spatially separated intratumoral biopsies per patient (Fig. 1c). Their analysis revealed tumor-wide NJs in all tested tumors. Additionally, also temporally conserved NJs were detected, as evidenced by their presence in the original as well as metastatic and recurrent post-treatment cancers (Fig. 1d).

Next, the authors sought to determine whether tumor-wide NJs could be detected on mRNA and protein level (Fig. 1e). Given the high ITH and poor immunotherapy outcomes in glioma patients, they focused their analysis on this tumor type. By analyzing publicly available RNA sequencing and mass spectrometry (MS) data sets, they were able to map neopeptides to 302 distinct public NJs. However, for neoantigens to be immunogenic, protein expression alone is not sufficient. The final protein products also need to be proteolytically processed, presented on HLA molecules, and recognized by T cells. To assess these questions, the authors first employed two algorithms to predict HLA presentation of antigens. By combining the algorithm-based predictions with the previously analyzed MS data, they selected 32 promising neopeptide-encoding NJs (NEJs) that were predicted to be presented on HLA-A*02:01, an allele highly prevalent across North America and Europe. Notably, most of these NEJs were also highly conserved in their spatially mapped glioma patient cohort.

The authors then investigated whether the peptides derived from these 32 NEJs could induce an HLA-dependent T cell activation (Fig. 1f). They stimulated donor-derived dendritic cells (DCs) with neoantigen peptides and co-cultured them with donor-derived CD8^+^ T cells (Fig. 1g). Two NEJ-derived antigens, NeoA_RPL22_ and NeoA_GNAS_, induced CD8^+^ T cell activation in this experiment. Through subsequent T cell receptor sequencing, they identified four T cell clones targeting these two NEJs. Using these TCR sequences, they engineered T cells expressing the corresponding TCRs and demonstrated HLA-dependent T cell activation by co-culturing peptide-pulsed T2 cells with the engineered T cells in the presence or absence of an anti-HLA antibody. Interestingly, they also detected T cells specific to NeoA_GNAS_ in one of three tested glioma patients.

To confirm the processing and presentation of NEJ-derived neoantigens, the authors transfected COS-7 cells with both whole NEJ-derived neoantigen transcripts and HLA-A*02:01. Mass spectrometry (MS) analysis showed that indeed HLA-neoantigen complexes were formed. These results demonstrate that the investigated NEJs are expressed, proteolytically processed, and presented on HLA molecules. This finding was underlined further by the successful activation of engineered T cells by transfected COS-7 cells. Additionally, by using MS the authors showed that the unmodified GBM cell line GBM115 expressed the NeoA_GNAS_ peptide.

Finally, the authors selected two clones (TCR_R3.0_ and TCR_G4.1_) to test T cell cytotoxicity against NEJ_RPL22_^+^ and NEJ_GNAS_^+^ GBM115 cells. Both clones showed significant target cell killing and the TCR_G4.1_ clone, which targets NEJ_GNAS_, also exhibited cytotoxicity against the GBM102 cell line and two melanoma cell lines. The HLA-dependency was further underlined by the observation that the addition of an anti-HLA antibody reduced the cytotoxicity. These findings provide compelling evidence that public NJ-derived neoantigens can indeed induce T cell responses and highlight the strong potential of NEJs as targets of tumor vaccines and engineered T cell therapies.

While the study demonstrates that some NJs occur tumor-wide and give rise to immunogenic neoantigens, the data also reveals differences in the prevalence of NJs between different cancer types and patients diagnosed with the same cancer. Due to the resource-intensive nature of multi-site biopsies and complex sequencing analyses, the authors sought biomarkers that correlate with the abundance of tumor-wide NJs. In GBM, they found that IDH1-mutant tumors showed significantly more tumor-wide NJs than IDH1-wildtype tumors, showing that subtypes of the same malignancy differ in their NJ abundance. Subsequently, the authors could link these differences to the differential expression of proteins involved in the splicing machinery such as CELF2, SNRPD2, and SF3A3. These findings provide a solid basis for selecting tumors with a high probability to exhibit a high prevalence of tumor-wide NJs based on a simple qPCR analysis of specific splicing factors.

In summary, this groundbreaking study demonstrates that public, spatially conserved NJs lead to neoantigen expression followed by HLA-dependent neoantigen presentation and CD8^+^ T cell activation. The authors identified two specific NEJs and two corresponding T cell clones that could potentially be used in tumor vaccines and transgenic TCR cell therapies across different cancer types. Furthermore, they established a robust methodology for discovering additional NEJs and selecting patients with a high likelihood of harboring abundant tumor-wide NJs in a cost-effective manner. In combination with the emergence of cheap mRNA vaccines, this could bring tumor vaccines to the next level. Despite the promising results of this study, the clinical application of immunotherapies has already taught us that tumor-specific T cell availability is often not sufficient to overcome immune-suppressive tumor microenvironments. Nonetheless, the ability to identify potent tumor antigens will bring us one step closer to also apply tumor vaccinations and immunotherapy combinations to resistant cancer types and patients.
